# Noncoding RNAs as novel biomarkers in pancreatic cancer: what do we know?

**DOI:** 10.2217/fon-2016-0253

**Published:** 2016-11-14

**Authors:** Maria C Previdi, Pietro Carotenuto, Domenico Zito, Rosantony Pandolfo, Chiara Braconi

**Affiliations:** 1 Division of Cancer Therapeutics, The Institute of Cancer Research, 15 Cotswold Rd, Sutton, SM2 5NG, UK; 2 The Royal Marsden NHS Trust London & Surrey, Downs Rd, Sutton, SM2 5NG, UK

**Keywords:** biomarker, lncRNA, miRNA, ncRNA, pancreatic cancer

## Abstract

Pancreatic cancer is an aggressive cancer of the digestive system, which is becoming a serious health problem worldwide. Overall survival for patients with pancreatic cancer is poor, mainly due to a lack of biomarkers to enable early diagnosis and a lack of prognostic markers that can inform decision-making, facilitating personalized treatment and an optimal clinical outcome. ncRNAs play an important role in pancreatic carcinogenesis. Here we review the literature on the role of ncRNAs as biomarkers in pancreatic cancer. We focus on the significance of ncRNAs as markers for early diagnosis, as prognostic biomarkers able to inform clinical management and as targets for novel therapeutics for patients with pancreatic cancer.

## Pancreatic cancer

Pancreatic cancer (PC) is one of the leading causes of death around the world [1]. Early diagnosis is frequently missed. Current imaging technologies have failed to detect early PC, and the absence of specific and sensitive biomarkers has limited the possibility of cost-effective screening for sporadic PC. About 9000 new diagnoses of PC are made in the UK each year, and >7000 of these patients live in England. Sadly, deaths from PC are as high as the diagnoses. Indeed, <5% of patients with pancreatic cancer are alive after 5 years, and even in patients amenable to surgical resection the 5-year survival rate is around 20% [2].

The most common type of PC arises from the pancreatic ductal cells and is named pancreatic adenocarcinoma (PDAC). In the past this was thought to be a homogeneous disease, but Collison *et al*. have provided evidence that supports the division of PDAC into subtypes according to molecular and genetic features. They identified a ‘classic’ subtype, characterized by high levels of expression of the adhesion-associated and epithelial genes, *AGR2* and *S100PBP*; a ‘quasi-mesenchymal’ subtype, which expresses the mesenchyme-associated genes twist family, *TWIST1* and *S100A2* at high levels and an ‘exocrine-like’ subtype, in which genes involved in the digestion process such as *REG3A* and *PRSS1* are highly expressed. These molecular subtypes have been shown to differ in clinical outcome and treatment response [3]. Recently, Bailey *et al*. delineated four new subtypes of PDAC based on data obtained from the RNA expression profiling of 456 PDAC patients: the ‘squamous’, the ‘progenitor’, the ‘immunogenic’ and the ‘aberrantly differentiated endocrine exocrine’, which are characterized by their various histopathological peculiarities and different overall survival (OS) [4]. Sequencing analyses have confirmed the heterogeneity of PDAC, indicating the potential for identifying independent subtypes with specific mutations in druggable genes [5].

It is well established that PDAC does not arise *de novo* but rather is preceded by noninvasive precursor lesions that undergo histologic and genetic progression culminating in invasive neoplasia. The most common premalignant precursors of PDAC are the pancreatic intraepithelial neoplasms (PaINs). There are different grades of PaINs, which are associated with different risks of invasive cancer. PaIN-1a lesions are characterized by a flat structure, while PaIN-1b lesions show a papillary architecture. Moderate to severe cytological abnormalities are present in PaIN-2 and -3 lesions, which exhibit a higher risk of malignancy. Other precursor lesions of PDAC include mucinous cystic neoplasms and intraductal papillary mucinous neoplasms (IPMN). IPMN is associated with an increased risk of malignancy, which is higher for the main duct and mixed forms (40–92%) but still present for the branch duct forms (15–25%) [6]. To date, the diagnosis of IPMN is made using imaging technologies; identifying biomarkers that are associated with malignant transformation is warranted if the clinical management of patients with IPMN is to improve.

Surgery represents the only treatment option which is potentially curative. Unfortunately, only 20% of PDACs are diagnosed at a resectable stage. Unresectability is mainly due to the encroachment of vascular and neurological structures, which may also be present in small tumors given the dense stromal reaction to PDAC. For this reason, >50% of curative resections result in positive resection margins, which account for the high risk of relapse [7]. Adjuvant treatment with single-agent chemotherapy (gemcitabine or fluorouracil) has proved beneficial with an increase in median survival from 15 to 23 months compared with observation alone [7]. However, OS after resection is still poor and clinical trials are ongoing to investigate if combination chemotherapy can improve the survival advantage in the adjuvant setting.

The prognosis of metastatic PDAC is dismal, with a median OS of about 6 months in the absence of any treatment. Systemic chemotherapy can improve this figure and has been the focus of clinical investigation over the last few years. Systemic treatment for advanced PDAC includes single-agent gemcitabine, a combination of two drugs or a combination of three drugs. The response rate and OS correlate with the number of drugs used, but unfortunately toxicity also significantly increases with the use of more drugs [2]. Increasingly, evidence is emerging that supports the use of second-line chemotherapy in patients who fail first-line treatment [2]. Thus, clinicians have the option of offering a triple combination as first line or adopting a sequential approach that may distribute the administration of the active drugs over the course of the disease, according to the outcome expected (neoadjuvant vs palliative setting).

The development of novel therapeutics can prompt the discovery of robust biomarkers, which can help to personalize treatment. Studies of protein-coding genes have failed to identify biomarkers of drug response in PDAC [8] with the exception of *hENT1*, which seems to predict the benefit from adjuvant gemcitabine [9]. The identification of predictive biomarkers in PC is complicated by the limited amount of material obtained from diagnostic cytology and the high level of heterogeneity within tumors. Indeed, efforts are being made to study ‘liquid biopsies’ in order to overcome these limitations [10].

## Noncoding RNA

The human genome includes genes that are translated into proteins (protein-coding genes) and genes that are transcribed into RNAs but lack the translation into proteins (ncRNAs). Protein-coding genes account for around 2% of the genome, the majority of which is instead represented by ncRNAs. For several decades ncRNAs were considered to be junk elements of the genome with no function. However, over the last few years there has been an increasing amount of evidence pointing to an essential regulatory role for ncRNAs. ncRNAs are now known to drive the biological complexity of vertebrates and to represent important players in evolutionary and developmental biology [11].

ncRNAs are classified as either sncRNAs, usually 20–30 nucleotides (nt) in length, or lncRNAs, >200 nt in length. The class of sncRNAs consists of different molecules involved in the mechanism of RNA interference, such as miRNAs, siRNAs, piRNAs and tiRNAs.

miRNA molecules are small ssRNAs consisting of 18–22 nt, which modulate the post-translational expression of several genes [12]. The biosynthesis of miRNAs starts in the nucleus and is completed in the cytoplasm. The first step is mediated by RNA polymerase II, and leads to the transcription of the primary miRNAs: long molecules characterized by a 5′ 7-methyl-guanylate cap and a 3′ poly(A) tail. These long molecules are several kilobases in length and are subsequently cleaved by the RNase III complex into 60–100 nt hairpin structures, called precursor miRNAs (pre-miRNA). The pre-miRNAs are transported from the nucleus to the cytoplasm by Exp5 proteins. In the cytosol, the pre-miRNAs are sliced by Helicase MOI into miRNA double strands. The two strands of each duplex are separated by helicases, and the nonfunctional strand is destroyed [12]. The mature functional strand is loaded into the RNA-induced silencing complex with hAgo, the catalytic component which promotes the base-pairing between the 5′-region of the miRNA and complementary target sites within mRNA – primarily the 3′ untranslated region [13]. The miRNA:mRNA base-pairing takes place through a short ‘seed-region’. If the miRNA:mRNA base-pairing is strong, the hAgo protein removes the mRNA poly-A-tail and exposes the molecule to exonucleases for degradation. If the base-pairing is less extensive the first direct effect is the inhibition of translation and then, probably, the mRNA is carried to the P-bodies and sequestered by ribosomes for degradation. Sometimes more than one miRNA is needed to bind the same mRNA and reduce its translation [13].

lncRNAs are sequences longer than 200 nt, transcribed by RNA polymerase II. Their genes are mainly located in intronic and intergenic regions, although in some cases they can overlap protein-coding genes. On the basis of their proximity to protein-coding genes lncRNAs can be classified as: antisense; sense; bidirectional; intronic; and intergenic lncRNAs. Most have the same structures as mRNAs, such as a 5′ cap and a 3′ poly(A) tail. lncRNAs can assume a secondary structure and can be localized in both the nucleus and the cytoplasm [14]. They can also undergo splicing at their 5′ and 3′ ends to generate circRNAs [15]. They are characterized by a paucity of introns and a low GC content, which accounts for their low level of expression within the cell. Intergenic and antisense lncRNAs were deemed to be more stable than other types of lncRNAs [16]. Several functions have been hypothesized for lncRNAs. They can act as cis- or trans-regulators of gene activity, as scaffold elements for chromatin-modifying complexes, as gene enhancers or as ceRNAs [17].

## miRNAs in PC

Despite their small size, endogenous miRNAs have been shown to have a remarkable effect on protein-coding gene expression. The deregulation of miRNA profiles has been implicated in several tumors. miRNAs can act as either tumor suppressors or oncogenes. Downregulation of tumor-suppressor miRNAs can promote the upregulation of genes involved in cancer progression, whereas overexpression of oncogenic miRNAs can elicit the downregulation of genes, which suppress tumor development. miRNA deregulation can be easily monitored by miRNA profiling and has offered new clues to understand pancreatic tumorigenesis.

The first evidence for miRNA deregulation in human PDAC came from expression-profiling studies, which identified several miRNAs aberrantly expressed in human PDAC in comparison to adjacent tissues, including miR-155, miR-21, miR-22/miR-222, miR-10 and miR-181 [18–21]. Identification of mRNA targets as key regulators of cell behavior confirmed the role played by miRNAs in the pancreatic carcinogenic process (Table 1).

## lncRNAs in PC

A novel role for lncRNA in PC has come to light in recent studies that have investigated the dysregulation of lncRNA in PC tissues compared with normal tissues (Table 2). These studies have highlighted higher levels of *H19* [37], *HOTAIR* [38], *HOTTIP* [39] and *MALAT-1* [40] in PC tissues. *PVT1* [41,42], *HULC* [43], *AF339813* [44], *LOC389641* [45] and *AFAP1-AS1* [46] also appear to be upregulated in PDAC. Conversely, *GAS5* and *ENST00000480739* were found to be downregulated in PDAC tissues compared with normal pancreatic tissues [47,48]. Nevertheless, the exact mechanism of action of these ncRNAs in PDAC remains to be fully elucidated. Gao *et al*. have suggested that the lncRNA *ROR* can act as a ceRNA in PC cells [49].

## Biomarkers in pancreatic adenocarcinoma

A biomarker is “a characteristic that is objectively measured and evaluated as an indicator of normal biological processes, pathogenic processes or pharmacologic responses to a therapeutic intervention”. There has been a growing effort to address the study of circulating biomarkers in PDAC, with the aim of identifying a noninvasive, reproducible, cost-effective biomarker that can be easily monitored over the course of the disease. To improve the clinical management of PDAC patients, a diagnostic biomarker that increases at the outset of the disease that can aid in early diagnosis, a prognostic factor that can address the need for adjuvant treatment and a predictive biomarker of response to chemotherapy are all needed. In addition, a blood-based marker would enable clinicians to follow tumor evolution, represent a surrogate marker for tumor heterogeneity and provide information from nonbiopsiable sites such as those with peritoneal carcinosis. To date, the only biomarker available for PDAC is Ca19-9. This antigen is overexpressed in 80% of patients with PC. However, its specificity for PDAC is poor and therefore its use as diagnostic biomarker is not recommended [58]. Ca19-9 has the potential to identify aggressive tumors and a decline in Ca19-9 after one cycle of chemotherapy may identify patients who would benefit from gemcitabine chemotherapy [59].

## ncRNAs as diagnostic biomarkers

miRNAs can circulate in the blood as free RNAs, which are bound to hAgo2, or included in exosomes; in either cases they are stable and they can be easily detected in patients’ biofluids [60]. Several authors have attempted to study the diagnostic value of circulating miRNAs in PDAC. The largest study looked at 409 patients with PC, 25 with chronic pancreatitis and 312 healthy participants [61]. 90% of PDAC patients were unresectable. In these cases whole blood was collected before the patients started chemotherapy, while in resectable cases sampling was undertaken before surgery. More than 700 miRNAs were tested in the discovery cohort, which included 141 cases of confirmed PDAC. Among the 38 miRNAs that differentiated patients with PDAC from those with chronic pancreatitis (n = 17) and healthy controls (n = 68) in the discovery cohort, 19 were validated in the training cohort (180 PDAC vs 199 healthy controls) and nine miRNAs were found to be consistently deregulated in the discovery, training and validation cohorts. These miRNAs were used to derive a diagnostic index based on miR-145, miR-150, miR-223 and miR-636. The sensitivity and specificity of this diagnostic miRNA panel were not superior to Ca19-9, but the combination of this index with Ca19-9 significantly enhanced the AUC when compared with Ca19-9 alone (AUC 0.93 vs 0.89) [61]. Interestingly, this index significantly correlated with the white blood cell, granulocyte and platelet count. Although this is not surprising, given that the analysis was performed on whole blood samples, it underlines the fact that the level of free miRNAs in the blood is much lower than the level detectable in cells. Therefore, great caution should be applied when choosing the source of the samples and the method of sample processing. Despite the fact that whole blood is easy to collect in clinical practice and using it avoids the need for extra steps and centrifugations, the information derived from whole blood may be different from the information derived from plasma and/or serum, which may better reflect the portion of free (or exosome-related) miRNAs and potentially be used as tumor surrogates. Li *et al*. identified miR-1290 in the serum of PDAC patients and observed that it had a higher diagnostic accuracy than Ca19-9 in their cohort (AUC 0.86 vs 0.77 in the group PDAC vs healthy controls) and was also expressed in tumor tissue, where it had prognostic significance [62]. Several other studies have found that circulating miRNAs or panels of miRNA found in the plasma and/or serum of PDAC patients have diagnostic potential and can improve upon the accuracy of Ca19-9 in diagnosis [63–65]. In selected cases salivary miRNAs were found deregulated in early PDAC [66]. However, the lack of concordance among these small studies represents the main challenge in implementing miRNA-based diagnostic tests in clinical practice. There are two likely reasons for this discordance: the normalization methods employed; the contribution of other medical conditions to the deregulation of circulating miRNAs. Standard reference genes used for the normalization of miRNA expression in tissues are not suitable for biofluid analysis [67], as this would imply the presence of whole cells in the biofluid. More recently, authors have attempted to normalize the expression of circulating miRNAs to one or a panel of other miRNAs. Unfortunately it is often the case that the expression of the miRNAs selected as reference genes is not consistent between samples, indicating that they may be involved in the pathogenetic mechanisms of the disease that the work is aiming to detect. For instance, miR-1228 was used as reference gene in the profiling of circulating miRNAs in liver cancer [68], and a few years later it was shown to modulate hepatocellular carcinoma (HCC) development having been shown to be altered in the sera of hepatocellular carcinoma patients [69]. Our view is that levels of circulating RNAs (like circulating tumor DNA or circulating proteins) must be normalized to the volume of biofluid, as our aim is to detect the amount of a certain element (miRNA in this case) in the fluid of an individual, which is not related to the number of cells present in the fluid. Indeed, miRNAs may derive not only from broken cells but also from a direct release of miRNAs from tissues. In addition, in the case of miRNAs that are released from dying circulating tumor cells it is reasonable to speculate that these miRNAs may be more stable in the circulation than other long RNAs. The other main limitation for the use of circulating miRNAs as diagnostic markers is the specificity for the tumor. Despite a growing amount of evidence supporting a link between the load of miRNAs in the tumor and the abundance of circulating miRNAs [70], we cannot forget that a number of other medical conditions may contribute to the profile of circulating miRNAs, such as liver and kidney injuries [71], sepsis [72], cardiovascular disease [73] and immunological disorders [74].

The expression of miRNAs in pancreatic tissue may also prove to be a useful marker when attempting to make a differential diagnosis in challenging cases. miR-196 is upregulated in PaIN-2/3 lesions and may help identify lesions with a higher risk of transformation [75,76]. miR-21 and miR-155 are upregulated in PDAC compared with adjacent tissue [19], and deregulation is also seen early on in IPMN, suggesting that these miRNAs may be considered as markers of transformation [77,78]. Studies designed to determine if the analysis of liquid from IPMN cysts can help in clinical decision-making are ongoing [79]. Of note, a recent analysis of miRNA expression in 55 endoscopic ultrasound-guided fine needle aspiration biopsies found that miR-21 and miR-155 levels could be used to differentiate malignant from benign lesions with a greater accuracy than standard pathology. However, comparison with other biomarkers (such as CEA) was not possible [80]. Another clinical challenge is making a differential diagnosis between PDAC and bile duct cancers. Collins *et al*. have identified miRNAs with disease-specific patterns of expression that may prove of benefit for such diagnostic purposes [81]. However, to date, none of these miRNA-based investigations have entered clinical practice as more validation is needed in large cohorts with comparable techniques.

## ncRNAs as prognostic biomarkers

miRNAs can modulate a plethora of protein-coding genes that are involved in the development, progression and metastatic spread of PDAC cells [22,82]. As a result, the potential for miRNAs to be used as prognostic markers in PDAC has been extensively studied. A number of reports have shown a correlation between miRNAs and OS in resected or inoperable PDAC, with miR-21 observed in multiple studies. Recently, Frampton *et al*. performed a meta-analysis of 20 studies, including >1500 patients [23]. This suggests that miR-21 may represent a good candidate to be validated in prospective cohorts. Thirteen studies concluded that miR-21 has a prognostic significance as a predictor of survival in PDAC. miR-21 was still associated with significantly poorer clinical outcomes even after the authors corrected for publication bias. In patients with resected PDAC, high expression of miR-21 could identify a risk of relapse and a response to adjuvant gemcitabine [23]. This suggests that miR-21 may represent a good candidate for validation in prospective cohorts. A statistically significant association was also observed for a few other miRNAs, even though these were investigated in a lower number of studies. Most notably, the downregulation of miR-34 was associated with a decrease in OS, consistent with previous evidence supporting the oncosuppressor role of this miRNA and its link with p53.

Based on published evidence, the assessment of miRNA expression in the tissue of resected PDAC may represent a feasible and promising marker for the identification of candidates amenable to adjuvant treatment. However, the assessment of miRNA expression may be more challenging in inoperable patients in which tissue availability is limited. Nonetheless, if the global deregulation of circulating miRNAs can be used as a surrogate measure of tumor features, as well as of the general comorbidities of a patient, it is likely to be a promising prognostic and predictive biomarker for patient selection [83–88]. We have proved that plasma levels of miR-21 can identify patients with particularly aggressive locally advanced PDAC who may not be suitable for a combination approach with chemoradiotherapy [70]. An increase in specific serum miRNAs was associated with resistance to lapatinib and capecitabine treatment in patients who have previously failed first-line chemotherapy [89], suggesting that miRNAs could be used as markers of different phases in PDAC evolution. Chemoresistance is a complex process that accounts for the low survival rate in PDAC patients. Cellular and stromal factors play a major role in drug resistance in PDAC. Indeed, miR-21 expression was shown to affect chemoresistance, not only by modulating cancer cell biology [24,90], but also by impacting on cancer-associated fibroblasts (CAF). Indeed, miR-21 is overexpressed both in PDAC cells and CAF within human PDAC, but only CAF-associated miR-21 was found to be associated with a response to fluorouracil in the RTOG 9704 trial [91].

Recent studies have shone a spotlight on a set of lncRNAs that could have a prognostic role in PC. *HOTAIR* is a lncRNA with pro-oncogenic activity in several tumors. The overexpression of *HOTAIR* was observed in PDAC human tissues associated with a poorer clinical outcome. *In vitro* data confirmed that this lncRNA is able to modulate cell proliferation and invasiveness [38]. *MALAT-1* is known to be an oncogenic lncRNA in other solid tumors [54]. Pang *et al*. looked at the expression of *MALAT-1* in 126 human PDAC tissues and observed that *MALAT-1* was overexpressed in most cases of PDAC, where it represented an unfavorable prognostic marker independent of clinical stage, tumor size, lymph node metastasis and distant metastasis [55]. In support of these findings, *MALAT-1* was found to be involved in the induction of a stem cell-like phenotype and in the regulation of cell migration and invasion in PDAC cells [40,56,57]. lncRNAs have been shown to be involved in the pathogenesis of PDAC. However, their value as clinical biomarkers has not been evaluated yet and future studies are warranted to address this topic.

## ncRNAs therapeutic targets

Recently miRNA delivery systems have been tested, alone and in combination with chemotherapeutic agents, to determine if it is possible to introduce miRNA therapeutics into biological systems in order to suppress PC. Liposomal nanoparticles carrying miR-34a or miR-143/miR-145 [92], and polymeric nanoformulations delivering miR-150 [93], proved able to inhibit pancreatic tumor growth *in vitro* and *in vivo*. Conversely, experiments using antisense oligonucleotides (ASOs) to target the main oncomiRs upregulated in cancer have confirmed the involvement of these oncogenic miRNAs in tumorigenesis. Administration of miR-21 ASOs [94] and miR-221 ASOs [95] resulted in a reduction in cancer cell proliferation, migration and chemoresistance in PC cells *in vitro* and *in vivo* in murine PC models. Interestingly, the co-administration of these ASOs significantly enhanced their effect [96]. Other studies have revealed that the co-administration of miRNA-mimics or anti-miRs and chemotherapeutics decrease the chemoresistance in PC. The administration of miR-205–gemcitabine conjugated micelles was shown to enhance the chemosensitivity of gemcitabine-resistant pancreatic cell lines and *in vivo* xenograft models, while reducing tumor proliferation and growth *in vitro* [97]. Transfection with miR-17-5p ASOs has also been identified as a potential approach for improving chemosensitivity to gemcitabine *in vitro* [98]. H19 regulatory sequences have been shown to have a potential role in pancreatic treatment, alone or in combination with gemcitabine [53]. *HOTTIP*, *HOTAIR* and *MALAT-1* knockdown increases the sensitivity of tumorigenic cells to gemcitabine *in vitro* and in *in vivo* xenograft models, suggesting that ncRNA-based therapeutics might be used in adjunction with conventional chemotherapeutic drugs to enhance their effect.

miRNA-based therapeutics have been investigated in humans and have been shown to be safe and efficacious in the treatment of hepatitis infection [99]. In animal models, inhibition or enforced expression of ncRNAs was shown to be feasible, safe and effective against cancer. Clinical trials of miRNA-based therapies in cancer patients are ongoing and it is likely that advances in technology will enable the selective delivery of ncRNAs (through nanoliposomal particles or viral vectors) to expand the current phase of investigation into miRNAs as therapeutics for the treatment of human disease. However, in all cases more effort is needed to identify and validate an appropriate ncRNA candidate in large and multiple studies.

## Conclusion

PDAC is a deadly disease that kills around 9000 people in the UK each year. Through the study of ncRNAs in PDAC we have gained an insight into the pathogenetic mechanisms underlying PDAC, opening the way to improved strategies for the clinical management of PDAC patients. A number of studies have highlighted the potential of ncRNAs as prognostic and predictive biomarkers, enabling a personalized clinical approach. If circulating miRNAs show some limitations as diagnostic markers due to a lack of specificity, we believe they may represent excellent prognostic and predictive biomarkers, which could inform upon the interaction between tumor and host. miR-21 expression, in particular, has consistently been found to correlate with clinical outcome, providing convincing evidence for taking this miRNA into the next step of prospective validation.

The use of ncRNAs alongside conventional therapeutic agents could become a promising approach for solving drug resistance in PC patients. Solid evidence has shown that miRNA-based therapeutics are feasible and safe in humans [99], and technological developments are likely to improve the administration of these therapeutics.

## Future perspective

The involvement of miRNA in the pathogenesis of PC is well supported. A growing amount of evidence points to the involvement of lncRNAs in pancreatic carcinogenesis. Long intergenic ncRNAs have received the greatest attention by the scientific community to date. However, recent findings suggest that other classes of lncRNAs, such as circular ncRNAs may represent promising mediators of PDAC development and progression [100]. Their role has been extensively studied in other cancers, and a deeper knowledge of their involvement in PDAC is vital in order to identify candidates for use as clinical biomarkers or targets of therapeutics.

There is now promising data that highlight the potential of miRNAs to act as biomarkers to inform the clinical management of PDAC patients. We believe that miRNAs may provide useful information in the assessment of patients’ prognosis and selection for treatment. As reviewed previously, the most interesting candidate so far is miR-21, measurement of which has the potential to stratify patients on the basis of disease aggressiveness and to identify the risk of relapse in early PDAC. Large prospective validation studies should be implemented in order to provide solid evidence for the potential incorporation of ncRNA-based biomarkers in clinical practice. The assessment of miRNAs in resected tissue specimens may be performed through a range of technologies including PCR-based technologies and *in situ* hybridization. While PCR-based technologies have the advantage of specificity, they pose the challenge of setting a threshold to be used in a prospective fashion. In contrast, *in situ* technologies enable visualization of the ncRNA signal and provide a semiquantitative measurement of ncRNA expression that can be used to stratify patients in analogy to standard-of-care technologies such as immunohistochemistry and FISH. Conversely, in advanced PDAC we believe that the assessment of circulating ncRNAs may represent a valuable tool. If miR-21 again represents a good candidate to assess patients’ prognosis, we suggest further studies to identify selected ncRNAs that predict response to different chemotherapeutic regimens. Preclinical evidence supports the involvement of ncRNAs in the modulation of the response to treatment and provides a rationale for studying ncRNAs as biomarkers of response in human patients. It is likely that the investigation of miRNAs in prospective large Phase III clinical trials represents the best and only strategy to define their role as prognostic and predictive biomarkers in PDAC, and, eventually, to improve the clinical management of PC patients.

**Table 1.** **miRNAs involved in the pathogenesis and progression of pancreatic cancer.**

**miRNA**	**Deregulation**	**Source**	**Biological process**	**Targets**	**Ref.**
miR-21/miR23a/miR-27a	Up	Human cell lines (PANC-1, MiaPaCa-2, LPc006, PLc167), xenograft models	Invasion, cell growth, proliferation, apoptosis	*PDCD4*, *BTG2*, *NEDDL4*, *PTEN*	[22–24]
miR-10b	Up	Human cell lines (PANC-1), human plasma	Invasion	*TIP30*, *p16*	[25,26]
miR-221/miR-222	Up	Human cell lines (BxPC-3, MiaPaCa-2, PANC-1, SW1990), human tissues	Proliferation, invasion, apoptosis	*MMP-2*, *MMP-9*	[27]
miR-155	Up	Human cell lines (MiaPaCa-2)	Apoptosis	*TP53INP1*	[28]
miR-193b	Down	Human cell lines (AsPC-1, PANC-1, CFPAC-1, Hs766T, SW1990, Mia PaCa-2, BxPC-3)	Apoptosis, cell-cycle progression, invasion	*MIR31HG*	[29]
miR-494	Down	Human cell lines (AsPC-1, PANC-1), xenograft models	Proliferation, migration, invasion, gemcitabine sensitivity	*FOXM1*, beta-catenin	[30]
Let-7	Down	Human tissues	Proliferation	*KRAS*, *MAPK*	[31,32]
miR-206	Down	Human cell lines (PANC-1, BxPC-3, MiaPaca-2, CFPAC-1, Colo357, Capan-1), human tissues, xenograft models	Invasion, migration, proliferation, cell-cycle progression	*KRAS*, *ANXA2*	[33]
miR-615-5p	Down	Human cell lines (BxPC-3, CFPAC-1, SW1990, PANC-1), human tissues, xenograft models	Migration, invasion, proliferation, tumor growth	*IGF2*	[34]
miR-219-1-3p	Down	Human cell lines (Capan-1, Capan-2, BxPC-3, PANC-1 and MiaPaCa-2), human tissues	Migration, proliferation, tumor growth	*MUC4*	[35]
miR-124	Down	Human cell lines (BxPC-3, CFPAC-1, SW1990, PANC-1), human tissues, xenograft models	Proliferation, invasion, metastasis	Rac1	[36]

**Table 2.** **lncRNAs involved in the pathogenesis and progression of pancreatic cancer.**

**lncRNA**	**Deregulation**	**Source**	**Biological process**	**Ref.**
*MIR31HG*	Up	Human cell lines (AsPC-1, PANC-1, CFPAC-1, Hs766 T, SW1990, MiaPaCa-2, BxPC-3)	Cell growth, apoptosis, cell-cycle progression, invasion	[29]
*LOC389641*	Up	Human cell lines (SW1990, AsPC-1, PANC-1, MiaPaCa-2, BxPC-3, Capan-2), human tissue	Progression	[45]
*AFAP1-AS1*	Up	Human cell lines (SW1990, PANC-1, MiaPaCa-2, BxPC-3, Capan-2, HPDE6), human tissues	Proliferation, migration, invasion	[46]
*HOTTIP*	Up	Human cell lines (SW1990, PANC-1, MiaPaCa-2, BxPC-3, Capan-2)	Cell progression, gemcitabine resistance	[39,50]
*PVT1*	Up	Human cell lines (AsPC-1)	Gemcitabine sensitivity	[41,42,51]
*HOTAIR*	Up	Human cell lines (PANC-1, Capan-2, SW1990, BxPC-3, MiaPaCa-2)	Radiosensitivity	[38,52]
*H19*	Up	Human cell lines (AsPC-1, PANC-1, CFPAC-1, SW1990, BxPC-3), human tissues	Metastasis, invasion, migration	[53]
*HULC*	Up	Human tissues	Metastasis vascular invasion	[43]
*MALAT-1*	Up	Human cell lines, (BxPC-3, CFPAC-1, CAPAN-1, SW1990, AsPC-1, PANC-1, HS766T), human tissues	Cell growth, migration, invasion	[40,54–57]
*ENST00000480739*	Down	Human cell lines (AsPC-1, BxPC-3, CFPAC-1, PANC-1, SW1990, HPNE), human tissues	Invasion	[48]
*GAS5*	Down	Human cell lines (BxPC-3, PANC-1, AsPC-1, and Hs766T), human tissues	Proliferation	[47]

**EXECUTIVE SUMMARY**

**Pancreatic cancer**

Pancreatic cancer is a deadly disease with poor clinical outcome.
Diagnosis is often late because of the lack of biomarkers.
Surgery is the only treatment that is potentially curative. Chemotherapy is beneficial, both single-agent and combination chemotherapy.

**ncRNAs in pancreatic cancer**

ncRNAs are encoded by genes that are transcribed into RNAs, but are not translated into proteins; they are classified as either sncRNAs or lncRNAs. Many ncRNAs are involved in the development of pancreatic adenocarcinoma.
The diagnostic and prognostic potential of ncRNAs has been investigated with promising results, but validation studies are needed before they can enter clinical practice.
Advances in ncRNAs delivery systems have increased the potential for ncRNA-based therapeutics.
